# Genetic profiling of healthy family members of breast and ovarian cancer patients in Estonia

**DOI:** 10.3389/fgene.2026.1814500

**Published:** 2026-07-07

**Authors:** Mikk Tooming, Kadri Rekker, Kadri Toome, Laura Roht, Piret Laidre, Olga Fjodorova, Hanno Roomere, Ülle Murumets, Ustina Šamarina, Sander Pajusalu, Riina Žordania, Kristi Tael, Eve Vaidla, Elvira Kurvinen, Neeme Tõnisson, Mihkel Ilisson, Peeter Padrik, Jaak Lehtsaar, Riina Kütner, Elen Vettus, Helen Vahar, Katrin Õunap, Tiina Kahre

**Affiliations:** 1 Department of Genetics and Personalized Medicine, Institute of Clinical Medicine, University of Tartu, Tartu, Estonia; 2 Department of Laboratory Genetics, Genetics and Personalized Medicine Clinic, Tartu University Hospital, Tartu, Estonia; 3 Department of Clinical Genetics, Genetics and Personalized Medicine Clinic, Tartu University Hospital, Tartu, Estonia; 4 Oncology and Haematology Clinic, North Estonian Medical Centre, Tallinn, Estonia; 5 Estonian Genome Center, Institute of Genomics, University of Tartu, Tartu, Estonia; 6 Gynaecology Department, West Tallinn Central Hospital, Tallinn, Estonia; 7 Haematology and Oncology Clinic, Tartu University Hospital, Tartu, Estonia; 8 Centre of Oncology, East Tallinn Central Hospital, Tallinn, Estonia

**Keywords:** breast cancer, cascade screening cancer syndromes, HBOC, healthy family members, molecular genetics of breast and ovarian cancer, NGS, ovarian cancer

## Abstract

**Background:**

Genetic testing for likely pathogenic/pathogenic variants (PV) in *BRCA1*, *BRCA2*, and other cancer-associated genes plays a critical role in the diagnosis, prognosis, and management of breast and ovarian cancer (BCOC). Extending testing to healthy family members (HFM) of affected individuals enables early prevention strategies and timely referrals for enhanced screening, thereby improving cancer risk management. This study aimed to characterize the demographic profile and genetic findings among HFMs of BCOC patients in Estonia within routine clinical practice.

**Methods:**

A retrospective analysis was conducted on 3,472 HFMs who underwent genetic testing. Demographic data were collected, and the presence of PVs was assessed. Statistical comparisons were made between individuals with and without known familial PVs, and between male and female participants, using descriptive statistics and proportion comparisons.

**Results:**

Of the 3,472 HFMs tested, 87.6% were female and 12.4% male, with a mean age of 41.1 ± 13.0 years. Notably, 78.6% were younger than 51 years, the typical age for initiating standard screening. PVs were identified in 683 individuals (19.7%). Among those with a known familial PV (n = 1,009), 41.8% were carriers, compared to 8.0% among those without a known familial PV (n = 2,408). Males were more likely to be tested when a familial PV was known (26.6%) than when it was not (6.6%), and 34.0% of tested males were PV carriers. PVs were found in 23 different genes, with *BRCA1/2* accounting for 58.4% of all PVs, followed by *ATM*, *BRIP1*, *CHEK2*, and *PALB2*.

**Conclusion:**

The findings highlight the value of genetic testing in identifying at-risk individuals among HFMs of BCOC patients. The predominance of *BRCA1/2* variants and the significant detection rate among younger individuals underscore the importance of early testing. The expansion of HFM testing in Estonia reflects increased public awareness and clinical integration of genetic risk assessment in cancer prevention strategies.

## Highlights

- Genetic testing for HFM increased substantially in Estonia from 2007 to 2023.
- The PV detection rate in HFM was 19.7%.
- *BRCA1/2* variants accounted for 58.4% of all detected PV.
- The NGS detection rate in HFM without familial PV was higher (8.3%) than targeted testing (6.9%).
- Males were referred for testing more often with a known familial PV (26.6% vs. 6.6%).

## Introduction

Genetic testing has become a crucial component in diagnosing breast and ovarian cancer (BCOC), especially for detecting hereditary breast and ovarian cancer (HBOC). Individuals with HBOC syndrome also face an increased risk of developing melanoma, pancreatic cancer, prostate cancer, and other types of cancer (Daly et al., 2026). The primary genetic risk factors for HBOC are likely pathogenic and/or pathogenic variants (PV) in the *BRCA1* (OMIM:113705) and *BRCA2* (OMIM:600185) genes. However, while some cases have a clear hereditary basis, others may be familial with no identified PV. Expanding genetic testing beyond *BRCA1* and *BRCA2* to other cancer-associated genes has refined risk assessment and management strategies.

Testing healthy family members (HFM) of individuals diagnosed with BCOC is a key component of proactive cancer prevention. It allows early detection of high-risk individuals, facilitating timely interventions such as enhanced surveillance, risk-reducing measures, and preventive treatments (Sessa et al., 2023; Daly et al., 2026). Additionally, genetic testing for HFM informs the risk of BCOC and aids in identifying susceptibility to other malignancies. Moreover, expanding the scope of genetic testing to include a broader range of genes increases diagnostic yield and can provide valuable information on the risk of various types of cancer, further enhancing early detection and personalized treatment strategies.

Various testing algorithms, schemes, and molecular methodologies are employed across European countries for HBOC and HFM (Marmolejo et al., 2021). The Estonian testing algorithm for HBOC and HFM primarily adheres to the guidelines established by the National Comprehensive Cancer Network (NCCN) and the European Society of Medical Oncology (ESMO). These guidelines provide a structured framework for genetic testing, risk assessment, and clinical management of individuals with a predisposition to HBOCs. Individuals with a significant family history should undergo genetic testing via multigene panels that include clinically validated HBOC-associated genes. Notably, relying solely on family history-based testing may fail to identify nearly half of HBOC syndrome gene carriers. Consequently, novel strategies are being developed to increase the identification of high-risk individuals and improve clinical outcomes (Sessa et al., 2023; Daly et al., 2026).

This observational study aimed to characterize the demographic characteristics and genetic testing outcomes of HFM referred for genetic testing from four major hospitals in Estonia between 2007 and 2023.

The primary objective was to characterize the population of HFM referred for genetic testing. The secondary objectives were (i) to evaluate the diagnostic yield of genetic testing by assessing the prevalence and distribution of PV, with particular emphasis on *BRCA1/2* and non-BRCA genes, and (ii) to assess trends in genetic testing practices over the study period. Given the observational and descriptive design of the study, no formal hypothesis testing was performed.

## Methods

### Participants and testing criteria

This nationwide retrospective observational study included cancer-free HFM of patients with BCOC, all of whom were managed according to routine clinical practice in Estonia during the study period. HFM were defined as individuals who were cancer-free at the time of genetic testing and were referred either because a PV had been identified in a BC/OC cancer relative or due to a strong family history. Individuals with a current or prior cancer diagnosis were not eligible for inclusion in this group. Because the study design was retrospective and based solely on existing clinical records, no longitudinal follow-up data were collected. There are currently no national guidelines for the genetic testing of HFM in Estonia. On the basis of the NCCN and ESMO guidelines (Sessa et al., 2023; Daly et al., 2026), Tartu University Hospital (TUH) has developed a publicly accessible guideline (JKL-165, 2006) that is also used and approved by the North Estonian Medical Centre (NEMC), which recommends genetic testing for hereditary BC and OC-associated gene variants as follows:

1. A hereditary BC and/or OC-causing PV has been previously detected in the family.
2. ≥2 primary BCs have been diagnosed in at least one individual among the first- or second-degree relatives in the family, regardless of age at diagnosis.
3. ≥2 individuals in the family have been diagnosed with primary BC.
4. At least one individual in the family has been diagnosed with OC, including OC of the fallopian tube or primary peritoneal cancer.
5. A first- or second-degree relative has been diagnosed with BC at ≤50 years of age or with triple-negative BC.
6. ≥3 individuals in the family have one of the following cancers (especially if diagnosed at a young age): pancreatic cancer, metastatic or high/very high-risk prostate cancer, sarcoma, adrenal cancer, brain tumour, endometrial cancer, thyroid cancer, kidney cancer, diffuse gastric cancer, dermatological manifestations and/or macrocephaly, or hamartomatous polyps in the gastrointestinal tract.
7. BC has been diagnosed in male(s) in the family.
8. The index BC/OC patient was diagnosed during the period when genetic testing was limited (e.g., single-gene or targeted mutation analysis was performed).

Eligible individuals were referred for molecular screening of cancer-associated PV by clinicians at four major hospitals in Estonia: TUH, NEMC, East Tallinn Central Hospital (ETCH), and West Tallinn Central Hospital (WTCH). Routine molecular testing was performed at the Genetics and Personalized Medicine Clinic at TUH between 2007 and 2023. Clinical data, such as sex, age, and familial cancer history, were gathered from test requisition forms or patient records provided by referring clinicians at the time of test ordering. All participants received standardized pre- and post-test genetic counselling delivered by certified clinical geneticists. Counselling covered the scope and limitations of testing, potential outcomes, and implications for patients and their families. For minors, testing and result disclosure followed national and international guidelines restricting reporting to variants relevant to childhood-onset conditions, with adult-onset findings withheld.

The cohort was divided into three groups: HFM with a known PV in the family, HFM without a known familial PV, and individuals who participated in different research projects in the Estonian Biobank (EstBB) cohort and needed genetic counselling and confirmation of a PV detected in a research setting.

Genetic testing was performed via targeted methods such as Sanger sequencing, arrayed primer extension (APEX; Asper Biotech Ltd.) microarray, multiplex ligation-dependent probe amplification (MLPA; MRC-Holland), or next-generation sequencing (NGS)-based approaches. To assess the distribution of PV, individuals were classified into three groups on the basis of the identified PV: “*BRCA1/2*”, including PV in the *BRCA1* or *BRCA2* genes, which represent high-penetrance genes with well-established HBOC clinical management recommendations; “other-NCCN”, encompassing PV in genes (*ATM, BARD1, BRIP1, CDH1, CHEK2, EPCAM, MLH1, MSH2, MSH6, PALB2, PMS2, RAD51C, RAD51D*, and *TP53*) listed in the National Comprehensive Cancer Network (NCCN) Genetic/Familial High-Risk Assessment: Breast, Ovarian, Pancreatic, and Prostate v3.2026 guidelines (Daly et al., 2026), excluding *BRCA1/2.* These genes are recognized by NCCN as having established or moderate evidence for hereditary cancer risk; and “non-NCCN,” comprising PV in genes (*FANCM, NBN, MUTYH, SDHA, MEN1, LZTR1, and HOXB13*) not included in the NCCN guidelines but associated with increased overall cancer risk. Individuals with no detected PV were categorized as “NEG”.

### Genetic testing

From 2007 to 2015, genetic testing primarily utilized an APEX genotyping DNA microarray, which included 66 of the most common clinically significant PVs in the *BRCA1*, *BRCA2*, *CHEK2*, and *RAD51C/D* genes. From 2008 to 2014, *BRCA1* and *BRCA2* whole-gene Sanger sequencing was employed to identify PV and target familial PV. Since 2009, MLPA of the *BRCA1* and *BRCA2* genes has been used to detect copy number variations (CNV). MLPA analysis was performed according to the manufacturer’s instructions.

In early 2014, NGS-based gene panels (TruSight Cancer, TruSight One; Illumina, Inc.) were introduced into regular clinical practice at TUH. Since 2015, the primary workflow for PV detection has involved initial testing for the most prevalent PV of *BRCA1* in Estonia, c.5266dup and c.4035del (Tamboom et al., 2010), followed by the NGS-based gene panel TruSight Cancer (94 genes) if the initial results are negative and subsequent MLPA analysis if needed. In 2021, the main *BRCA1* PV detection was discontinued, and only NGS gene panel testing was performed. In 2018, the NGS gene panel was updated from TruSight Cancer to TruSight Hereditary Cancer (113 genes), TruSight One (4,800 genes), and TruSight One Expanded (6,700 genes), all by Illumina, Inc. During the study period, for cases with known familial PV, Sanger sequencing of the respective gene was employed to detect the PV.

### Sequencing data analysis and data interpretation

Raw sequencing data from FASTQ files were aligned to the hg19 reference genome via BWA (Li and Durbin, 2009). Bioinformatic processing and variant calling were performed via the Genome Analysis Toolkit (GATK) tools (McKenna et al., 2010). Variants were annotated via an in-house variant annotation pipeline via SnpSift (Ruden et al., 2012), ANNOVAR software (Wang et al., 2010), RefSeq (O’Leary et al., 2016), dbSNP (Sherry et al., 2001), ExAC (Karczewski et al., 2017), gnomAD (Chen et al., 2024), ClinVar (Landrum et al., 2014), and OMIM (Amberger et al., 2009). CNV detection was carried out via CoNIFER (Krumm et al., 2012) or DeCon (Fowler, 2022) software. ACMG guidelines (Richards et al., 2015) were used for variant classification, and variants were described according to HGVS nomenclature (den Dunnen et al., 2016). Variants of uncertain significance (VUS) are not reported, and no automated re-evaluation system is in place. Patients with negative results may be re-assessed after 3–5 years, particularly when hereditary cancer is suspected, and can return for a follow-up consultation with a clinical geneticist, during which their results may be re-evaluated based on updated evidence.

### Statistical analysis

The data were analysed via R Studio 2024.12.0 and Python 3.9 with “pandas” and “statsmodels” libraries. The data were assessed via the chi-square test to evaluate the prevalence of PV among different groups and the Welch t-test to compare the mean age of the HFM with a known PV in the family, comparing the HFM without a known familial PV. A multivariate logistic regression analysis was conducted to evaluate factors associated with PV detection. Predictor variables included known familial PV status, gender, age at testing, the number of relatives with cancer and testing era (≥2015 vs. ≤2014). A multivariate logistic regression model with an added intercept was fitted using maximum likelihood estimation to obtain adjusted odds ratios and 95% confidence intervals. A p-value of <0.05 was considered statistically significant for all analyses.

## Results

A total of 3,472 HFM samples were analysed, in which genetic testing was conducted as part of routine clinical practice. The majority of samples originated from TUH (2,932; 84.5%), the main hospital providing genetic counselling and molecular testing services in Estonia, followed by NEMC (460; 13.2%), ETCH (59; 1.7%), and WTCH (21; 0.6%).

The demographic distribution revealed a predominance of females (3,042; 87.6%) compared with males (430; 12.4%). Most participants were in the 31–40 years age group (1,078 individuals, 31.0%). The general characteristics of the cohort are summarized in Table 1.

**TABLE 1:** Overall study participants’ demographics, family history of cancer, and overview of pathogenic/likely pathogenic variants.

​	HFM group (n = 3,472)											
Females (%)	3,042 (87.6%)											
Males (%)	430 (12.4%)											
Mean age of testing (±SD)	41.1 ± 13.0											

Number of relatives with cancer in the family	Unknown				1		2		3		≥4	
Individuals within a group (%)	89 (2.6%)				1,077 (31.0%)		1,080 (31.1%)		661 (19.0%)		565 (16.3%)	
Mean number of relatives with cancer in the family	2.3											

Age groups	≤30		31–40		41–50		51–60		61–70		≥71	
Gender	F	M	F	M	F	M	F	M	F	M	F	M
Individuals (n) (%) n = 3,472	611 (17.6%)	115 (3.3%)	966 (27.8%)	112 (3.2%)	830 (23.9%)	94 (2.7%)	368 (10.6%)	61 (1.8%)	201 (5.8%)	35 (1.0%)	66 (1.9%)	13 (0.4%)
Individuals within age group (%) n = 3,472	726 (20.9%)		1,078 (31.0%)		924 (26.6%)		429 (12.3%)		236 (6.8%)		79 (2.3%)	
Number of individuals with PV in age group (%)	201 (27.7%)		185 (17.2%)		149 (16.1%)		76 (17.7%)		41 (17.4%)		18 (22.8%)	

HFM with a known PV in the family (n = 1,009 individuals) (mean age of testing 38.4 ± 14.6)												
​	Females n = 741 (73.4%)				Males n = 268 (26.6%)				Total HFM with a known PV in the family (n = 1,009)			
HFM with one PV	281				132				413			
HFM with two PV	7				2				9			
HFM with PV	288 (38.9%)				134 (50.0%)				422 (41.8%)			
NEG (%)	453 (61.1%)				134 (50.0%)				587 (58.2%)			

HFM without a known familial PV (n = 2,408 individuals) (mean age of testing 42.2 ± 12.1)												
​	Females n = 2,248 (93.4%)				Males n = 160 (6.6%)				Total HFM without a known familial PV (n = 2,408)			
HFM with one PV	177				12				189			
HFM with two PV	4				0				4			
HFM with PV	181 (8.1%)				12 (7.5%)				193 (8.0%)			
NEG (%)	2,067 (91.9%)				148 (92.5%)				2,215 (92%)			

HFM, healthy family members; F, female; M, male; PV, pathogenic/likely pathogenic genetic variants; NEG, negative results.

**FIGURE 1:**
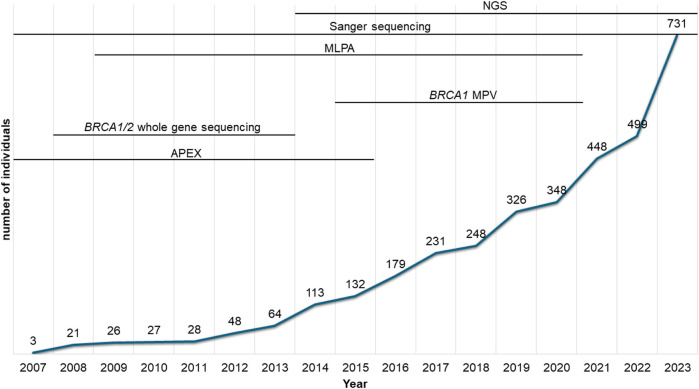
Number of individuals tested via various molecular diagnostic methods from 2007 to 2023. The lines represent the years of utilization of different methods in routine clinical practice. APEX, Array primer extension; *BRCA1* MPV, *BRCA1* main pathogenic variant analysis (c.5266dup and c.4035del); MLPA, multiplex ligation-dependent probe amplification; NGS, next-generation sequencing.

The mean age at testing was 41.1 years (±13.0); the average age for females was 41.2 (±12.7) years, and that for males was 40.5 (±15.1) years. Overall, 78.6% of the HFMs referred for molecular testing were younger than 51 years, and 52.0% were younger than 41 years. Over the study period, the mean age of individuals tested decreased from 44.6 years in 2007 to 42.6 years in 2023. The mean age at testing in the HFM group was 38.4 years (±14.6) for individuals with known familial PV and 42.2 years (±12.1) for those without. This difference was statistically significant (p < 0.05). The number of tested HFM per year increased dramatically over the study period. A substantial increase in testing occurred from 2013 to 2014 (+77%), and the increase from 2022 to 2023 (+46%) is particularly noticeable (Figure 1).

The family history of cancer within the cohort was diverse. A total of 1,077 individuals (31.0%) reported having one cancer-affected relative, followed closely by 1,080 individuals (31.1%) with two affected relatives. Additionally, 661 individuals (19.0%) reported three affected relatives, whereas 565 individuals (16.3%) reported four or more relatives with cancer. HFM reported on average, 2.3 cancer-affected relatives. Only a small fraction of participants (89; 2.6%) were categorized as having an unknown family history of cancer. This group comprised individuals in whom BC/OC was suspected on their first oncological examination, and genetic testing was ordered, but subsequent investigations did not confirm the presence of cancer, and/or individuals had very high anxiety about hereditary cancer.

Overall, 683 carriers of PV associated with elevated cancer risk were identified, yielding a diagnostic efficacy of 19.7%, indicating that approximately one in five individuals tested carried a PV. A total of 533 PVs were detected in females, and 150 PVs were detected in males. The PV reported in the age groups indicates that the highest prevalence of PV is found in the ≤30 years age group (27.7%). The prevalence of PV in the ≤30 years age group was significantly different (p < 0.05) from that in the overall combined age group. The diagnostic yield during period 2007–2014 was 12.1% (40/330) and during 2015–2023 was 20.0% (630/3142). The details are summarized in Table 1.

### Individuals with known familial PV

Among the 3,472 individuals who underwent genetic testing, 1,009 (29.1%) had known PV in their family. This group included 741 females (73.4%) and 268 males (26.6%). Notably, seven females and two males carried two PVs. Overall, 422 individuals (41.8%) in this group were found to have a total of 431 PVs. Specifically, 288 females (38.9%) and 134 males (50.0%) were identified as PV carriers (Table 1). PV in *BRCA1/2* constituted the majority, with 277 cases (64.3%) of all PV in this cohort (177 females and 100 males). This was followed by other-NCCN PVs, 137 cases (31.8%), and non-NCCN PVs, 17 cases (3.9%; Table 2).

**TABLE 2:** Pathogenic/likely pathogenic genetic variants were identified among the tested healthy family members.

Reported PV in HFM with a known PV in the family (total PV = 431)			
​	PV in females n = 295 (68.4%)	PV in males n = 136 (31.6%)	Total PV = 431
*BRCA1/2* PV (%)	177 (60.0%)	100 (73.5%)	277 (64.3%)
Other-NCCN PV (%)	102 (34.6%)	35 (25.7%)	137 (31.8%)
Non-NCCN PV (%)	16 (5.4%)	1 (0.7%)	17 (3.9%)

Reported PV in HFM without a known familial PV (total PV = 197)			
​	PV in females n = 185 (93.9%)	PV in males n = 12 (6.1%)	Total PV = 197
*BRCA1/2* PV (%)	75 (40.5%)	5 (41.7%)	80 (40.6%)
Other-NCCN PV (%)	86 (46.5%)	7 (58.3%)	93 (47.2%)
Non-NCCN PV (%)	24 (13%)	0	24 (12.2%)

HFM, healthy family members; Other-NCCN, Genes reported in the National Comprehensive Cancer Network guideline Genetic/Familial High-Risk Assessment: Breast, Ovarian, Pancreatic, and Prostate v3.2026 (excluding *BRCA1* and *BRCA2*) (*ATM, BARD1, BRIP1, CDH1, CHEK2, EPCAM, MLH1, MSH2, MSH6, PALB2, PMS2, RAD51C, RAD51D, TP53*); Non-NCCN, Genes not listed in the National Comprehensive Cancer Network guideline Genetic/Familial High-Risk Assessment: Breast, Ovarian, Pancreatic, and Prostate v3.2026 (*FANCM, NBN, MUTYH, SDHA, MEN1, LZTR1, HOXB13*); PV, Pathogenic/Likely Pathogenic Genetic Variants; NEG, negative results.

**FIGURE 2:**
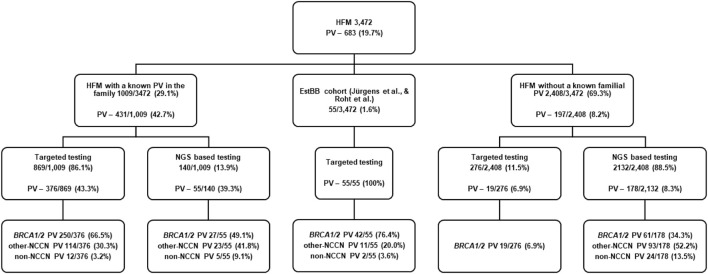
Diagram of the study cohort. This figure illustrates the distribution of pathogenic/likely pathogenic variants (PVs) among 3,472 healthy family members (HFMs) from four major hospitals treating breast and ovarian cancer patients in Estonia. The analysis compared PV detection rates in two groups, “HFM with a known PV in the family” and “HFM without a known PV in the family”, via different molecular diagnostic methods. Targeted testing methods included Sanger sequencing, APEX (Arrayed Primer Extension Microarray), and MLPA (Multiplex Ligation-dependent Probe Amplification). Next-generation sequencing (NGS) methods included TSC (TruSight Cancer), TSHC (TruSight Hereditary Cancer), TSO (TruSight One), TSOE (TruSight One Expanded), and whole-exome sequencing (WES). In the HFM with a known PV in the family group, among the 869 individuals tested with targeted methods, 43.3% had PV, primarily in *BRCA1/2* (66.5%), followed by the other-NCCN (30.3%) and non-NCCN (3.2%) genes. Among the 140 individuals tested with NGS, 39.3% had PV, with a greater proportion of other NCCN genes (41.8%) than with targeted testing. In the HFM without a known familial PV group, 276 individuals underwent targeted testing, identifying 6.9% PV, all in *BRCA1/2*. Among the 2,132 individuals tested with NGS, 8.3% had PV (34.3% *BRCA1/2*, 52.2% other-NCCN, and 13.5% non-NCCN). Additionally, in the previous studies by Roht et al. and Jürgens et al. and the Estonian BioBank (EstBB) cohort (55 individuals), PVs were identified in 76.4% of the *BRCA1/2*, 20% of the other-NCCN, and 3.6% of the non-NCCN genes. The other-NCCN includes genes listed in the National Comprehensive Cancer Network (NCCN) Genetic/Familial High-Risk Assessment: Breast, Ovarian, Pancreatic, and Prostate v3.2026 guidelines (*ATM, BARD1, BRIP1, CDH1, CHEK2, EPCAM, MLH1, MSH2, MSH6, PALB2, PMS2, RAD51C, RAD51D, TP*53). Non-NCCN refers to genes not included in the guidelines (*FANCM, NBN, MUTYH, SDHA, MEN1, LZTR1, HOXB13*).

Predominantly, 869 out of 1,009 individuals with known familial PV were tested via targeted methods. A total of 376 PVs were identified, representing 43.3% of the tested group. In total, 250 PVs (66.5%) were found in the *BRCA1/2* genes, 114 PVs (30.3%) in the other-NCCN genes, and 12 PVs (3.2%) in the non-NCCN genes. Additionally, 140 individuals underwent testing via an NGS panel due to an extensive cancer history in both parents’ pedigrees. PV was detected in 55 individuals, representing 39.3% of this group (Figure 2).

### Individuals without known familial PV

The cohort without identified familial PV (n = 2,408) consisted of 2,248 females (93.4%) and 160 males (6.6%). Four females carried two PVs. In total, 193 individuals (8.0%) in this group were found to have PV, including 181 females and 12 males (Table 1).

A total of 197 PVs were identified in this cohort, with 185 (93.9%) detected in females and 12 (6.1%) in males. In this cohort, 80 patients (40.6%) had *BRCA1/2* PV, 93 patients (47.2%) had other-NCCN PV, and 24 patients (12.2%) had non-NCCN PV (Table 2).

Individuals without known familial PV were investigated primarily via NGS panels. Among the 2,132 individuals assessed, 178 PVs were detected (8.3%). Among these genes, 61 (34.3%) were *BRCA1/2* PV genes, 93 (52.2%) were other-NCCN genes, and 24 (13.5%) were non-NCCN genes. Additionally, 276 individuals were assessed via targeted methods, leading to the identification of 19 PVs (6.9%), all of which were *BRCA1/2* PVs (Figure 2).

Fifty-five individuals were part of earlier research projects by Roht et al. (Roht et al., 2023), which focused on the Lynch syndrome spectrum in Estonia, and Jürgens et al. (Jürgens et al., 2022), who studied participants with breast cancer PV. These individuals, from the Estonian Biobank cohort (EstBB), tested positive for PV in genes associated with HBOC and/or Lynch syndrome in the research setting. All these individuals were referred for routine genetic counselling, family history collection, and verification of detected PV, and they were subsequently included in our study. Among EstBB individuals, *BRCA1/2* PV was reported in 42 individuals (76.4%), other-NCCN group PV was identified in eleven individuals (20.0%), and non-NCCN PV was reported in two individuals (3.6%). In the multivariate logistic regression analysis, individuals with a known familial PV exhibited a 6.25-fold increase of PV detection compared with those without a familial PV. Gender, age at testing, and family history were all statistically significant confounders (p < 0.05) (Table 3).

**TABLE 3:** Multivariate logistic regression analysis results for PV detection in individuals with and without a known familial PV, adjusted for age, gender, number of affected relatives and testing era.

Predictor	Adjusted OR	95% CI (lower)	95% CI (upper)	p-value
Known PV in family (yes vs. no)	6.25	5.14	7.60	<0.001
Gender (F vs. M)	0.73	0.57	0.94	0.013
Age at testing (per year)	0.99	0.99	1.00	0.031
Number of relatives with cancer (per relative)	1.15	1.08	1.23	<0.001
Testing era (≥2015 vs. ≤ 2014)	1.29	0.89	1.86	0.174

### Molecular findings

**FIGURE 3:**
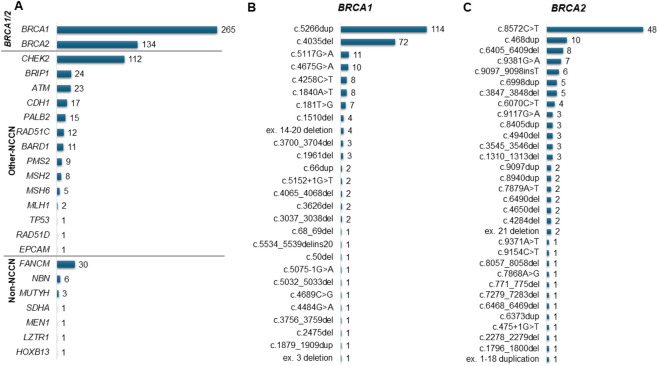
**(A)** Comprehensive list of genes with pathogenic/likely pathogenic variants (PVs) associated with increased cancer risk. The other-NCCN includes genes listed in the National Comprehensive Cancer Network (NCCN) Genetic/Familial High-Risk Assessment: Breast, Ovarian, Pancreatic, and Prostate v3.2026 guidelines (*ATM, BARD1, BRIP1, CDH1, CHEK2, EPCAM, MLH1, MSH2, MSH6, PALB2, PMS2, RAD51C, RAD51D, TP*53). Non-NCCN refers to genes not included in the guidelines (*FANCM, NBN, MUTYH, SDHA, MEN1, LZTR1, HOXB13*). **(B)** Detailed list of pathogenic/likely pathogenic variants in the *BRCA1* gene identified in HFMs (transcript NM_007294.4). **(C)** Detailed list of pathogenic/likely pathogenic variants in the *BRCA2* gene identified in HFMs (transcript NM_000059.4).

The most frequent PV was identified in the *BRCA1* gene, reported 265 times (7.6%; one in 13 individuals), followed by the *BRCA2* gene in 134 cases (3.9%; one in 26 individuals). The most common *BRCA1* PV, NM_007294.4 (BRCA1):c.5266dup, p. (Gln1756Profs74), was found in 114/265 individuals (43.0%), whereas NM_007294.4 (BRCA1): c.4035del, p. (Glu1346Lysfs20) was detected in 72/265 individuals (27.2%). These two *BRCA1* variants accounted for 186 out of the 683 (27.2%) cases of PV. The most frequent PV in the *BRCA2* gene, NM_000059.4 (BRCA2): c.8572C>T, p. (Gln2858*), was identified in 48/134 individuals (35.8%). Other *BRCA1/2* PVs and their detailed distributions are illustrated in Figure 3. Notably, one 19-year-old individual harbouring PV in both *BRCA1* and *BRCA2*, inherited from the mother, passed away from ovarian cancer at the age of 46.

PVs in genes from the “other-NCCN” list were identified in 241 cases (6.9%; one in 14). In the other-NCCN group, the most frequently reported PVs were *CHEK2,* with 112 (16.4%), *BRIP1,* with 24 (3.5%), and *ATM,* with 23 (3.4%) cases. Additionally, PVs were detected in the *CDH1*, *PALB2*, *RAD51C*, *BARD1*, *TP53*, and *RAD51D* genes, accounting for 57/683 (8.3%) of the total. Lynch syndrome-associated PVs (*PMS2*, *MSH2*, *MSH6*, *MLH1*, and *EPCAM*) were found in 25 patients (3.7%).

PV in genes of the “non-NCCN” group was reported in 43 cases (1.2%; one in 81). The non-NCCN group included PV in the following genes: *FANCM* 30 (4.4%), *NBN* 6 (0.9%), and *MUTYH* 3 (0.4%). Additionally, single variants were reported for *SDHA*, *MEN1*, *LZTR1*, and *HOXB13* (0.6%). The prevalence and frequency of all PVs are detailed in Figure 3A.

### Molecular testing in males

**FIGURE 4:**
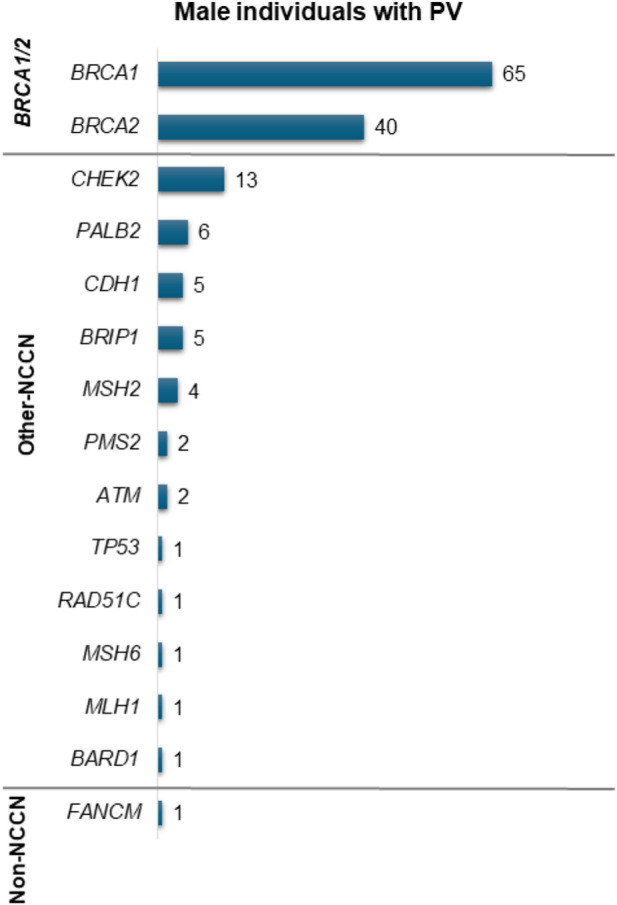
Distribution of males with pathogenic or likely pathogenic variants. Two males harbored PVs: one in *CHEK2/PALB2* and one in *BRCA2/CHEK2*. The other-NCCN includes genes listed in the National Comprehensive Cancer Network (NCCN) Genetic/Familial High-Risk Assessment: Breast, Ovarian, Pancreatic, and Prostate V3.2026 guidelines (*ATM, BARD1, BRIP1, CDH1, CHEK2, EPCAM, MLH1, MSH2, MSH6, PALB2, PMS2, RAD51C, RAD51D, TP*53). Non-NCCN refers to genes not included in these guidelines (*FANCM, NBN, MUTYH, SDHA, MEN1, LZTR1, HOXB1*3).

Overall, 430 males underwent genetic testing, with 148 (34.4%) identified as carriers of PV, totaling 150 PVs. Males were more likely to seek testing when a known familial PV was present (Table 1; Figure 4). In the known familial PV group, males comprised 26.6%, compared to just 6.6% without a known familial PV, indicating greater uptake. In males, PV was most frequently detected in *BRCA1* in 65 (15.1%) individuals, followed by *BRCA2* in 40 (9.3%) individuals and *CHEK2* in 13 (3.0%) individuals. PV in *ATM, BARD1, BRIP1, CDH1, FANCM, MLH1, MSH2, MSH6, MUTYH, PALB2, PMS2, RAD51C,* and *TP53* accounted for a total of 30 (7.0%) cases. Two PVs involving the *CHEK2* gene were identified in two male subjects (Figure 4).

## Discussion

In Estonia, HFM testing is centred at the Genetics and Personalized Medicine Clinic at TUH because it is the leading clinic providing genetic and molecular testing services. While clinicians of various specialties in Estonia can order genetic tests, the oncologists participating in our study focused primarily on testing cancer patients. However, it is essential to emphasize that testing HFM is equally crucial for effective cancer prevention and management. Over the 17-year study period, the number of HFM patients who underwent genetic testing increased significantly from three individuals to 731 (Figure 1). Stratification of the cohort by testing era demonstrated a higher diagnostic yield in the NGS period (≥2015; 20.0%) compared with the pre NGS era (≤2014; 12.1%). However, this difference reflects the combined effects of technological heterogeneity, including broader gene coverage and differing analytical approaches, as well as substantial referral and selection bias. In earlier years, genetic testing was largely restricted to highly selected families and limited gene targets, whereas later periods were characterized by broader eligibility criteria and the use of multigene NGS panels.

Studies have reported that the cascade testing rate for HBOCs and Lynch syndrome is often less than 50% (Srinivasan et al., 2020). Predictors of genetic testing uptake include higher personal or familial cancer risk and referrals from healthcare providers (Scott et al., 2020; Sarki et al., 2022), younger age (Ladd et al., 2020), and female sex (Sanz et al., 2010; Childers et al., 2018; Sarki et al., 2022). As we also observed in our study, these factors significantly influence the likelihood of individuals undergoing genetic testing, highlighting the importance of targeted strategies to improve testing rates and ensure early detection. In older age groups, the cancer risk associated with PV has likely already manifested, leading these individuals to seek testing as cancer patients. However, our findings indicate that continued attention is necessary for those over 71 years, as standard screening is typically discontinued in this age group (US Preventive Services Task Force, 2024; Sõeluuring.ee, 2026). Notably, in our cohort, PV was detected in 22.8% of individuals in this age group. Overall, 78.6% of HFM patients referred for molecular testing were younger than the standard screening age of 50 years for mammography and planned prostate cancer screening (Eestis on kanda kinnitamas uued sõeluuringud | Tervisekassa, 2024; Sõeluuring.ee, 2026). Testing HFM below 50 is crucial because the cumulative risk of BC in *BRCA1* and *BRCA2* carriers increases after age 30. For *BRCA1* mutation carriers, the cumulative risk for OC increases around age 40, whereas for *BRCA2* mutation carriers, it increases around age 50 (Kuchenbaecker et al., 2017). PV in *BRCA1* and *BRCA2* have well-established HBOC clinical management recommendations, including intensified breast surveillance, risk-reducing surgery, and prostate cancer surveillance in male carriers, as outlined in NCCN and ESMO guidelines (Sessa et al., 2023; Daly et al., 2026). Additionally, our study revealed that younger patients are more likely to participate in genetic testing than older patients are, which is in accordance with the findings of the study by Ladd et al. (2020). Multivariate logistic regression analysis comparing individuals with and without a known familial PV showed that female gender and older age at testing were each modestly associated with lower odds of PV detection, whereas individuals with known familial PV and a higher number of affected relatives was positively associated with the likelihood of identifying a PV (Table 3). Notably, individuals with a known familial PV underwent testing at a younger mean age (38 years) compared with those without such a family history (42 years). Therefore, earlier testing would help direct PV carriers to surveillance programs, introduce preventive measures, and manage hereditary cancer risks. In Estonia, NEMC has launched a pilot project for systematic follow-up of PV carriers, involving alternating mammography and breast magnetic resonance imaging every 6 months, along with gynaecological ultrasound. Additionally, TUH, ETCH, and other hospitals have established surveillance programs through dedicated breast cancer centres.

### Possible founder mutation in *BRCA2* and recurring PVs in *BRCA1*

Among the entire cohort, *BRCA1* and *BRCA2* were the genes with the highest frequency of PV. This aligns with previous studies highlighting the predominance of *BRCA1/2* variants in HBOC syndromes (Marmolejo et al., 2021; Henkel et al., 2023). The most prevalent *BRCA1* PVs detected in our HFM cohort were c.5266dup and c.4035del (Figure 3). These *BRCA1* PVs were also the main PVs in Estonia reported in one previous work on *BRCA1/2* testing of BC patients by Tamboom et al. (2010) and the EstBB cohort by Leitsalu et al. (2021). The *BRCA1* c.5266dup PV is a frequent variant in several European countries, including Greece, Hungary, Latvia, Lithuania, Montenegro, Poland, Russia, Serbia, and others (Hamel et al., 2011). The *BRCA1* PV c.5266dup and c.4035del are consistently prevalent in Baltic cohorts. In the Latvian *BRCA1/2* cohort by Loza et al. (2021), c.5266dup occurred in 57.2%, and c.4035del occurred in 38.8% of cases. In the Lithuanian cohort by Janavičius et al. (2014), c.4035del was found in 49.0%, and c.5266dup was found in 28.6% of cases. The most prevalent *BRCA2* PV in our study, c.8572C>T, has been described in the literature related to BCOC (Breast et al., 2021). This *BRCA2* variant was previously identified in the EstBB cohort (Leitsalu et al., 2021; Jürgens et al., 2022), where it was the most frequently detected *BRCA2* variant. It has also been reported in two unrelated families in Lithuania (Janavičius et al., 2014) and Latvia (Loza et al., 2021). Given its rarity in Latvia and Lithuania, and other populations, according to the gnomAD database (Chen et al., 2024), this variant may represent a founder mutation within the Estonian population. However, its detection in unrelated populations, including reports from Italy and Korea indicates that its historical origin may be more complex than a strictly local founder event and needs further investigations (Marroni et al., 2004; Ryu et al., 2019).

The high detection rate of 42.7% for known familial PV using targeted testing highlights the effectiveness of these methods when familial PV is known. Targeted testing typically employs Sanger sequencing of the known PV. This approach is cost-effective, delivers faster results, and avoids unnecessary analysis. However, NGS could also remain valuable in the known PV group, as it could identify additional PVs, particularly when there is a history of cancer on the other side of the family, which targeted testing may not detect. In individuals without prior familial PV, the overall PV incidence was lower (8.2%) than that in those with known familial PV. Notably, the distribution of PV was more even between the *BRCA1/2* (40.6%) and other-NCCN genes (47.2%). This finding supports the expansion of genetic testing beyond *BRCA1/2* in individuals suspected of having hereditary cancer, as over half of all PVs in this group were found in other-NCCN and non-NCCN genes. The NCCN and ESMO guidelines (Sessa et al., 2023; Daly et al., 2026) also recommend the use of NGS multigene panel testing. The higher detection rate of PV through NGS (8.3%) than through targeted testing (6.9%) further underscores the utility of NGS in cases where a familial mutation is unknown.

### Prevalence of other cancer-associated genes

The other-NCCN gene group contributed 35.3% of all reported PVs identified in our HFM cohort. In the routine clinical workflow, we must not limit our focus solely to *BRCA1* and *BRCA2* when testing for hereditary BCOC syndromes. Broad NGS-based gene panel testing allows the identification of PV in other BCOC-related genes listed in the NCCN and ESMO guidelines (Sessa et al., 2023; Daly et al., 2026). These guidelines include well-studied genes extensively described in association with BCOC, Lynch syndrome, pancreatic cancer, colorectal cancer, and other cancer syndromes. Numerous studies, expert panel working groups, and guidelines in the scientific literature have corroborated their importance in relation to cancer (Breast et al., 2021; Marmolejo et al., 2021; Henkel et al., 2023; Sessa et al., 2023; Daly et al., 2026). If HFM patients harbour PV, they may be eligible for management and risk-reduction strategies in accordance with established clinical guidelines, supporting targeted prevention and personalized care for at-risk individuals. For several moderate-risk NCCN-listed genes identified in this study, including *PALB2*, *CHEK2*, and ATM, guideline-based recommendations are available but differ from those for *BRCA1/2* (Daly et al., 2026).

In Estonia, the standard clinical workflow also reports incidental findings of PV in high-risk genes not listed in the NCCN and ESMO guidelines (Sessa et al., 2023; Daly et al., 2026) because alterations in these genes, such as *MEN1*, may be significant for other cancer types and could substantially impact genetic counselling within families. Among the other genes mutated, the most common were *FANCM*, *NBN,* and *MUTYH*. Additionally, PVs were detected in the *SDHA*, *MEN1*, *LZTR1*, and *HOXB13* genes. *FANCM* has been associated with specific types of BC, such as estrogen receptor-negative BC (Breast et al., 2021). Despite these findings, *FANCM* is not currently included in the guidelines (Sessa et al., 2023; Daly et al., 2026), and its association needs further evaluation to understand the full implications of including *FANCM* in clinical guidelines. Some of the reported genes are associated with various autosomal-dominant diseases, including *SDHA* in pheochromocytoma/paraganglioma (OMIM:600857), *MEN1* in multiple endocrine neoplasia (OMIM:613733), *LZTR1* in schwannomatosis type 2 (OMIM:600574), and *HOXB13* in prostate cancer (OMIM:604607). However, BCOC can be part of the clinical spectrum for some of these syndromes. Additionally, individuals with a monoallelic PV in *NBN* (OMIM:602667) or *MUTYH* (OMIM:604933), which are linked to recessive tumour predisposition syndromes, do not have an elevated tumour risk compared with the general population. Although non-NCCN genes were included due to their presence on diagnostic panels and potential relevance for genetic counseling, their clinical actionability varies widely, and many represent moderate-risk or emerging associations with limited guideline-based management recommendations (Daly et al., 2026). However, these variants may be important to consider in genetic counselling dependent on the family history and partner testing for family planning. Henkel et al. (2023) evaluated various gene panels for germline analysis in a cohort of 6,941 individuals who met the criteria for genetic testing of HBOCs in Germany. Their findings indicated that using only the HBOC core panel, which included 14 genes, resulted in a diagnostic yield of 10.8%. However, this targeted panel failed to detect 66 variants located outside the core panel. The study concluded that restricting genetic testing to HBOC-related genes alone could be limiting, potentially depriving patients and their relatives of opportunities for cancer surveillance, early detection, and risk reduction.

### Genetic testing in males

Our findings indicate that most HFMs referred for genetic testing were females (87.6%). Heald et al. (Heald et al., 2024) also reported a high proportion of females (76%) in the studied cohort and low uptake of genetic testing in males (Bednar et al., 2020; Sarki et al., 2022). Potential reasons for this include the tendency of healthcare providers to overlook the necessity of testing male family members for HBOC-related PV. Moreover, family members may not always encourage men to pursue genetic counselling; additionally, men might demonstrate lower health awareness (Sanz et al., 2010). Furthermore, we found that males were more likely to participate in testing when familial PV was known, comprising 26.6% of that group. In contrast, they constituted only 6.6% of the group in which a familial PV was unknown. It is well known that men with *BRCA2* PV face an absolute risk of developing early-onset prostate cancer of up to 61% and a lifetime risk of BC of up to 7%. In our cohort, the mean age at testing for males was 40.5 years. According to the National Comprehensive Cancer Network (NCCN) guidelines, prostate cancer surveillance is recommended for male *BRCA1/BRCA2* carriers at the age of 40 years (Daly et al., 2026). In 2024, Estonia initiated a feasibility study on risk-based prostate cancer screening for men aged 50 and older, following recommendations from the European Association of Urology (Van Poppel et al., 2021; Sõeluuring.ee, 2026). Identifying male carriers of PV facilitates their timely integration into standard cancer screening. Raising awareness of the need for male genetic testing in cases of HBOC in the family will help reduce the sex gap in PV cascade screening.

### Genetic testing practices and trends in Estonia

In Estonia, medical doctors mainly follow the NCCN guidelines. The only publicly available Estonian guideline developed by TUH (JKL-165, 2006) refers to the NCCN and ESMO guidelines (Sessa et al., 2023; Daly et al., 2026). This finding suggests that NGS can be used to analyse genes associated with HBOC and other cancer-related syndromes rather than focusing solely on core HBOC genes such as *BRCA1/2*. PV in the *BRCA1*, *BRCA2*, and other *non-BRCA* genes is associated with an increased risk of breast, ovarian, fallopian tube, gastric, melanoma, peritoneal, prostate, and pancreatic cancers and different types of cancer (Sessa et al., 2023; Daly et al., 2026). The implementation of NGS in routine diagnostics remains essential, particularly when no familial PV has been identified. Advances in bioinformatics now enable the detection of various genetic alterations, including single-nucleotide variants and copy number variations, streamlining workflows and reducing reliance on multiple testing methods. This integration helps shorten the turnaround time of the results. Nevertheless, a key challenge of NGS is the complexity of interpreting variants of unknown significance, which can complicate clinical decision-making.

One of the main limitations of this study is the heterogeneity of genetic testing methods over time, together with limited information available from HFM referral forms. As a result, some individuals who initially tested negative using earlier targeted methods were not retested when more comprehensive NGS-based approaches became available. This likely contributed to under-diagnosis of hereditary cancer syndromes and incomplete genetic risk stratification during the early years of the study. Importantly, this study assessed diagnostic yield rather than clinical outcomes, and no longitudinal follow-up data were available to evaluate the impact of genetic testing on cancer incidence, surveillance adherence, or clinical management in HFM. Therefore, increased variant detection rates observed in later testing periods should not be interpreted as evidence of improved clinical outcomes. Nevertheless, in the context of HFM, expanded NGS-based testing provides a more comprehensive assessment of inherited cancer risk and enables increased identification of individuals eligible for established guideline-based surveillance and preventive strategies. Thus, the benefit observed in this study relates to improved genetic risk identification and the potential for subsequent clinical action. An additional limitation is the marked gender imbalance in the cohort, with a predominance of female participants, which may limit the generalizability of the findings, particularly with respect to hereditary cancer risk assessment and surveillance implications in male family members. A strength of our study is the observed trend of increasing uptake of cascade genetic testing, as shown in Figure 1. This surge in genetic testing might be driven by the public disclosure in 2013 by Angelina Jolie, who revealed that she carried a *BRCA1* PV. This announcement, known as the “Jolie effect”, increased awareness in the general population and led to a substantial rise in genetic testing and understanding of hereditary cancer risk (Evans et al., 2014).

In conclusion, integrating HFM with PV into risk reduction strategies enables early identification and prevention, potentially lowering cancer incidence. The decreasing mean age at testing in our cohort reflects improved referrals and awareness. Despite the increasing accessibility of NGS, targeted testing for known familial PV remains the primary approach for HFM. However, NGS may be preferable in cases where a familial PV is unknown or when additional cancer predisposition genes are of concern. Careful patient selection is essential to optimize diagnostic utility. The increasing use of genetic testing among HFM patients in Estonia underscores increasing awareness and acceptance of hereditary cancer risk assessment, reinforcing its critical role in precision medicine and public health initiatives.

## Data Availability

Datasets from Tartu University Hospital are not publicly available due to protocol and local regulations. The genetic sequencing data presented in the publication originate from clinical testing. Requests to access them should be directed to Mikk Tooming, email: mikk.tooming@kliinikum.ee
